# Molecular understanding of postharvest flower opening and senescence

**DOI:** 10.1186/s43897-021-00015-8

**Published:** 2021-08-24

**Authors:** Xiaoming Sun, Meizhu Qin, Qin Yu, Ziwei Huang, Yue Xiao, Yang Li, Nan Ma, Junping Gao

**Affiliations:** Beijing Key Laboratory of Development and Quality Control of Ornamental Crops, State Key Laboratory of Agrobiotechnology, Department of Ornamental Horticulture, College of Horticulture, China Agricultural University, Beijing, 100193 China

**Keywords:** Flower opening, Petal expansion, Petal senescence, Phytohormones, Environmental factors, Regulatory mechanisms

## Abstract

Flowers are key organs in many ornamental plants, and various phases of flower development impact their economic value. The final stage of petal development is associated with flower senescence, which is an irreversible process involving programmed cell death, and premature senescence of cut flowers often results in major losses in quality during postharvest handling. Flower opening and senescence are two sequential processes. As flowers open, the stamens are exposed to attract pollinators. Once pollination occurs, flower senescence is initiated. Both the opening and senescence processes are regulated by a range of endogenous phytohormones and environmental factors. Ethylene acts as a central regulator for the ethylene-sensitive flowers. Other phytohormones, including auxin, gibberellin, cytokinin, jasmonic acid and abscisic acid, are also involved in the control of petal expansion and senescence. Water status also directly influences postharvest flower opening, while pollination is a key event in initiating the onset flower senescence. Here, we review the current understanding of flower opening and senescence, and propose future research directions, such as the study of interactions between hormonal and environmental signals, the application of new technology, and interdisciplinary research.

## Introduction

By definition, flowers are of central importance in floral crops, and the processes of flower opening and senescence are key determinants of their ornamental quality and economic value (Srikanth and Schmid 2011). In angiosperms, flower opening is important for reproduction, often through the presentation of petals to attract animals that promote cross-pollination (van Doorn and Kamdee 2014). Once pollination is accomplished, petals complete their biological function and quickly enter the phase of senescence (Ketsa et al. 2001). Petal senescence can be affected by fruit development, which is necessary for plants to complete reproductive development. During postharvest shipping and storage, ethylene, and other harmful gases, can cause undesired opening of flowers and early senescence of petals, resulting in serious postharvest losses (Ma et al. 2005, 2006). Therefore, strategies to ensure desired flower opening and to delay petal senescence are important for optimizing postharvest flower quality and are topics of great interest in flower research.

In the current review, we summarize the molecular mechanisms known to be involved in flower opening and senescence, their regulation by phytohormones and environmental factors, and suggest future research directions.

## Molecular control of postharvest flower opening

### Flower opening is driven by cell division and expansion

Flower opening depends on petal expansion and movement and is a complex process, typically involving flower bud swelling, opening, bending outwards, folding, and finally development into a mature flower (Rizzo and Harampolis 2013). These floral organ movements lead to different types of flower opening, which vary between species and varieties. For instance: flower opening in poppy (*P. somniferum*) involves the release of mechanical constraints (Reid and Evans 1986); the loose flower structure of *C. persicum* is caused by petal expansion (Rolland-Lagan et al. 2003); and in Asiatic lily (*Lilium*), flower opening is mainly promoted by a change in the angle between the midrib and pedicel (Bieleski et al. 2000a). Differences in the rate, direction and anisotropy of cell expansion in different petal regions can all contribute to different types of flower opening (Rolland-Lagan et al. 2003). Petal expansion depends on a change in cell number and size, and flower opening is often accompanied by both cell division and cell expansion (Pei et al. 2013; van Doorn and Kamdee 2014). In rose (*Rosa hybrida*), flower opening driven by both cell division and cell expansion involves irreversible petal movement that is manifested in a transition from an entangled position to a horizontally expanded position (Yamada et al. 2009). In contrast, *E. grandiflorum* flower opening results from reversible asymmetric expansion of cells on the abaxial and adaxial side of petals (Ryo et al. 2016). Flower organ growth begins after formation of the flower primordium and all cells enter the stage of continuous division. Changes in the timing or rate of continuous cell division can result in changes in cell number, which in turn lead to changes in organ size (Gonzalez-Carranza et al. 2012; Czesnick and Lenhard 2016). Before flowers open, the petals complete organogenesis and most petal cells stop dividing, except for a limited number of the cells that maintain the capacity for division (Martin & Gerats, 1993; Huang and Irish 2016). After this time, petal expansion is mainly driven by increasing cell size (Ma et al. 2008).

Cell expansion depends on synergistic changes in cell wall metabolism, cell turgor and cytoskeletal reorganization (Zonia and Munnik 2007) and is a highly complex process. The spatially controlled synthesis, degradation, and restructuring of cell wall components results in the coordinated control of cell growth, a process that is strictly regulated by developmental and environmental factors (Cosgrove 2005). The irreversible ductility, or plastic deformation, of the cell wall is a key factor in the expansion of petal cells (Yamada et al. 2009), and to this end cell wall localized proteins, such as expansins and xyloglucan endotransglycosylase/hydrolases (XTHs), are involved in cell wall relaxation (van Sandt et al. 2007; Harada et al. 2011). Current models propose that expansins interfere with hydrogen bonds between polysaccharide molecules in the wall, thereby causing it to relax and making it more amenable to turgor-driven extension (Cosgrove 2015).

Cell turgor is the driving force for cell expansion, where a transmembrane osmotic potential gradient generated by intracellular solute accumulation causes water to move across the plasma membrane through aquaporins, and accumulate in the vacuole (Zonia and Munnik 2007). In *Gentiana kochian* and *G. kochiana*, flower opening mainly depends on the differential turgor-driven cell expansion on the two sides of the petal (Gookin et al. 2003; van Doorn and van Meeteren 2003).

Another key factor in cell expansion is remodeling of the cytoskeleton, an intracellular network composed of microtubules and microfilaments. Microtubules have been shown to contribute to the morphology of petal epidermal cells and to play a role in regulating the anisotropic shape of petals and the formation of cone cells (Panteris et al. 1994). The direction of cell expansion largely depends on the arrangement of microtubules. In the cell expansion stage, periplasmic microtubules at the outermost periphery of the protoplast are arranged perpendicular to the axis of cell elongation, and when these microtubules are arranged diagonally or longitudinally with the cell elongation direction, cell expansion is inhibited (Lloyd 2011). Thus, it is thought that microtubules control the direction of cell expansion through deposition of cellulose microfibrils, but the exact mechanism is still unclear (Wang and Jiao 2020).

### Phytohormones control flower opening

Multiple phytohormones have a critical function in regulating organ development (Durbak et al. 2012) and affect flower opening through a series of sequential steps. As summarized below, it has been found that ethylene, gibberellin (GA), auxin, abscisic acid (ABA), jasmonic acid (JA) and brassinolide (BR) are all involved in flower opening.

#### Ethylene

Ethylene has been shown to promote flower opening in many ornamental plants, such as gladiola (*Gladiolus*), carnation (*D. caryophyllus*), petunia, orchid (*Phalaenopsis*), rose, wintersweet (*Chimonanthus praecox*), and others (Serek et al. 1994; Tang and Woodson 1996; Bui and O'Neill 1998; Jones and Woodson 1999; Ma et al. 2005; Sui et al. 2015). In the case of wintersweet, an exogenous ethylene treatment can rapidly promote flowers to reach the stage of full bloom, while exposure to 1-methylcyclopropene (1-MCP), an ethylene action inhibitor, significantly delays or inhibits flower opening, causing flowers to fail to open completely or remain partially open until wilting (Sui et al. 2015). Interestingly, ethylene can also promote flower opening in rose, but significantly reduces petal size by inhibiting petal cell elongation, suggesting that the effect of ethylene on rose flower opening may depend on the petal movement rather than the cell expansion (Ma et al. 2008).

**Fig. 1:**
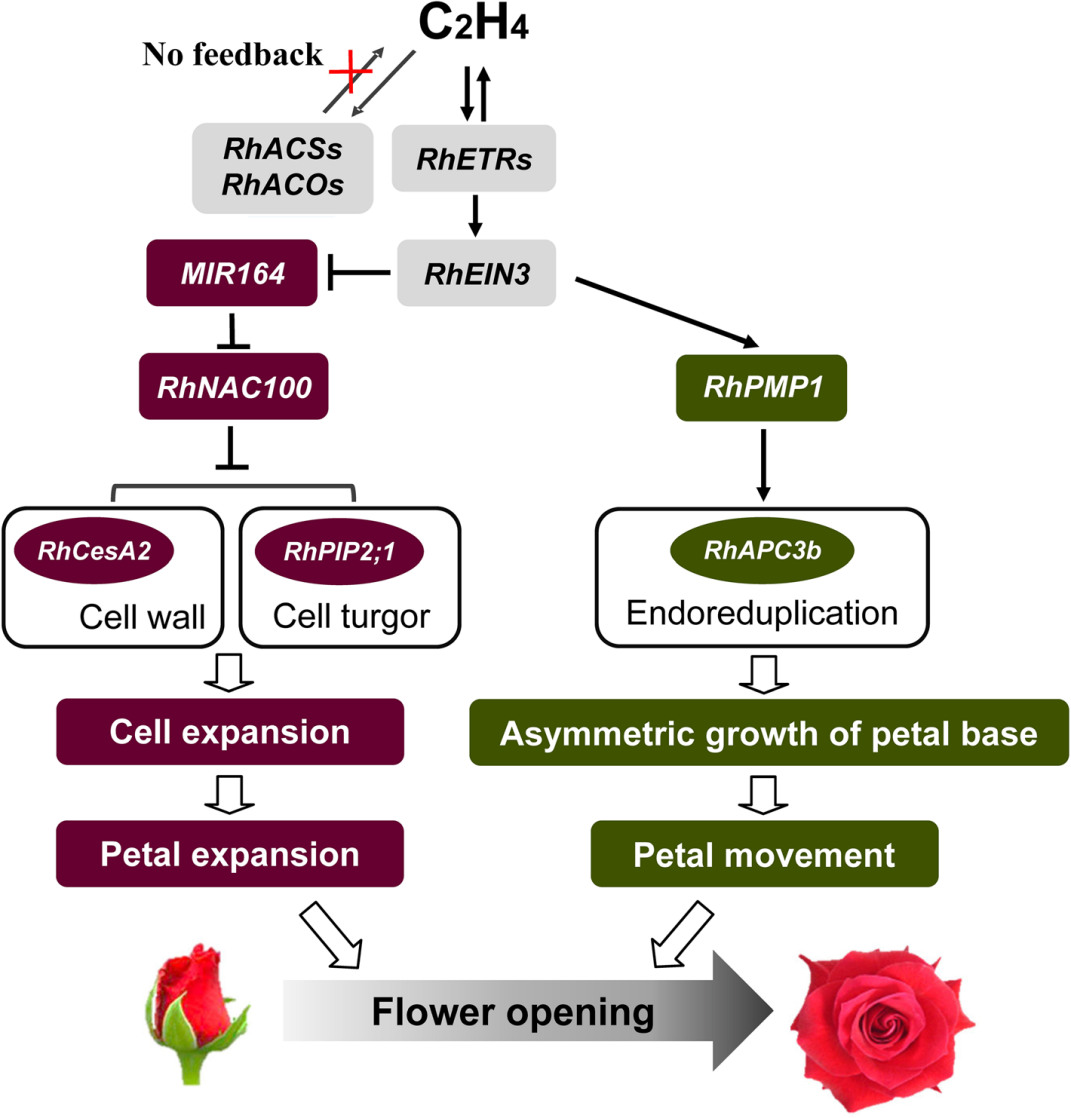
Proposed model of rose flower opening regulation by ethylene

The mechanisms by which ethylene functions have been widely studied. Ethylene regulation of flower opening in rose (Fig. 1) first involves perception of ethylene rather than ethylene synthesis (Ma et al. 2005, 2006). That is to say, the effect of ethylene on rose flower opening is not through a positive feedback regulation of ethylene biosynthesis, but through up-regulation of genes encoding ethylene receptors. Of these, *RhETR3* is the most important receptor gene in the mediation of ethylene signaling to promote flower opening (Ma et al. 2005, 2006, 2008; Tan et al. 2006). Among the five flower organs (sepals, petals, stamens, pistil and receptacle) in rose, the pistil is the first to respond to the ethylene signal (Ma et al. 2008). Notably, RhPIP2;1, a member of the aquaporin family, is involved in ethylene-regulated cell expansion of rose petals. Silencing of *RhPIP2;1* results in a significant increase in the number of abaxial sub-epidermal (AbsE) cells in the petals, reduces the irregular shape of AbsE cells, and an obvious decrease in the length and width of the petals (Ma et al. 2008). Similarly, *RhPIP1;1* is involved in the regulation of ethylene-induced petal cell expansion and its silencing results in decreased petal size and cell area, as well as reduced fresh weight (Chen et al. 2013). Another key gene involved in ethylene-regulated rose flower opening is the NAC transcription factor *RhNAC100*. Interestingly, it’s expression is controlled via microRNA164-dependent post-transcriptional regulation. *RhNAC100* regulates petal expansion by modulating the expression of cell expansion-related genes, such as *RhPIP2,1* and the cellulose synthase gene *RhCesA2*. Thus, ethylene regulates petal cell expansion by fine-tuning the microRNA164-RhNAC100 module, thereby promoting flower opening (Pei et al. 2013). Recently, the rose *PETAL MOVEMENT-RELATED PROTEIN 1* (*RhPMP1*) transcription factor gene was identified as a direct target of ETHYLENE INSENSITIVE 3 (EIN3) which is a key transcription factor downstream of ethylene signaling. *RhPMP1* could increase *RhAPC3b* expression and specifically activates cell endoreduplication in the parenchyma on the adaxial side of the petal base. The expression of cell expansion genes, such as *RhBXL*, *RhPE* and *RhRCI3*, was upregulated and induced asymmetric growth of the petal base. Therefore, a current model involves ethylene regulating a RhEIN3-RhPMP1-RhAPC3b transcriptional cascade to promote petal movement and flower opening (Cheng et al. 2021) (Fig. 1).

#### Auxin

Auxins, such as indoleacetic acid (IAA) and naphthaleneacetic acid (NAA), promote flower opening and their exogenous application leads to an enhanced rate and angle of *Iris* flower opening (van Doorn et al. 2013), while treatment with auxin inhibitors has the opposite effect. Similar results have been reported for water lily (*N. lotus*), where treatment with an auxin inhibitor suppressed flower opening (Ke et al. 2018). During petal expansion and growth, auxin-related genes are up-regulated to promote cell elongation. Transcriptome analysis of chrysanthemum (*C. morifolium*) flowers showed that auxin-related genes, including signal transduction genes or transcription factors, regulate petal expansion through the TEOSINTEBRANCHED 1, CYCLOIDEA and PCF (TCP) transcription factor genes (Wang et al. 2017). Auxin response factor 8 (ARF8) was also shown to interact with BIGPETALp (BPEp) to modulate petal expansion by restricting mitotic growth in the early stages of petal development and cell expansion in the later stages (Varaud et al. 2011).

#### Gibberellins

The gibberellins (GA) class of phytohormones, of which there are several bioactive forms, function as promoters of flower opening in several species, including *I. nil*, *Gerbera hybrida*, and *L. sinuatum* (Steinitz and Cohen 1982; Raab and Koning 1987; Li et al. 2015). In *G. hybrida*, GA_3_ promotes the expansion of ray floret petals and an increase in cell size (Li et al. 2015), while in *C. sativus*, GA_9_ is naturally produced in the ovaries and moves to the sepals and petals, where it is converted into bioactive GA_4_, which has been shown to regulate organ growth (Lange and Lange 2016). GA concentration also increases sharply in *Gaillardia* petals when the corolla grows rapidly, and decreases significantly at later stages (Kening 1985).

GA regulates flower opening mainly through crosstalk with ethylene, and in rose, the ethylene-inhibited transcription factor RhNF-YC9 participates in regulating the expansion rate and size of petal cells by mediating GA synthesis. Accordingly, silencing *RhNF-YC9* was observed to reduce expression of gibberellin acid biosynthetic gene *RhGA20ox* and increases gibberellin acid catabolic gene *RhGA2ox* transcripts level, resulting in a decreased rate of petal expansion (Chen et al. 2020). Crosstalk between GA and ethylene also regulates petal expansion by modulating *DELLA* gene expression and protein stability. Ethylene treatment induces the expression of the rose *DELLA* gene *RhGAI1* via EIN3. Silencing of *RhGAI1* promotes cell expansion in rose petals. RhGAI1 directly binds to the promotor of the cell wall cellulose synthesis gene *RhCesA*2 and repress its expression, thereby inhibiting flower opening (Luo et al. 2013).

#### Other hormones

Jasmonic acid (JA) also functions as a general promoter of flower opening, and a reduction in JA biosynthesis in *A. thaliana* was observed to cause delayed petal growth and flower opening (Ishiguro et al. 2001). The JA signal-insensitive tomato *jai1–1* mutant has delayed flower opening (Niwa et al. 2018), and in *E. grandiflorum*, treatment with the JA analog methyl jasmonate (MeJA) significantly accelerates petal growth and flower opening, involving increased expression of cell wall modification associated genes (e.g. the expansin genes *EgEXPA2*, *EgEXPA3* and the XTH gene *EgXTH1*) (Ochiai et al. 2013). JA has also been found to inhibit lateral tepal movement in *Iris* flowers and to prevent flower opening (van Doorn et al. 2013).

Finally, the brassinosteroid (BR) class of phytohormones promote flower opening, and in *Gerbera*, preferentially stimulate cell elongation in the middle and basal regions of petals to enhance petal growth (Huang et al. 2017).

### Environmental factors influence flower opening

Plant growth and development is fundamentally affected by environmental factors, and those that are known to influence flower opening include water status, temperature, the dark-light cycle, carbohydrates and nutrients. As outlined below, each of these have been shown to adversely affect inter-related aspects of flower opening, hormone balance and cell growth (van Doorn and Kamdee 2014).

#### Water relations

**Fig. 2:**
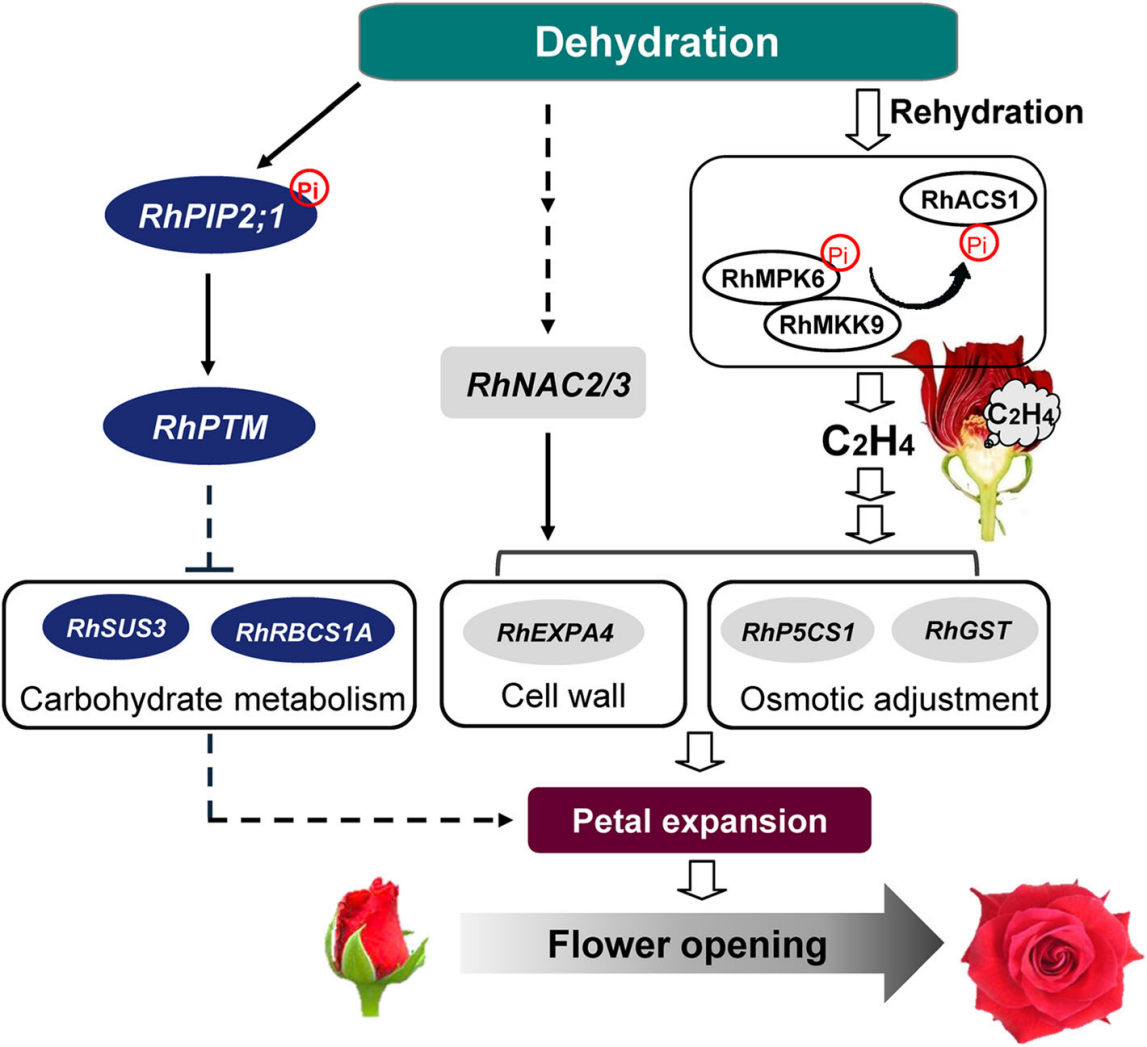
Proposed model of controlled postharvest rose flower opening

Maintaining an adequate water status is critical for ensuring high quality in open cut flowers and for prolonging their vase life. Under postharvest conditions, cut flowers often suffer from water deficit stress. Dehydration often leads to air embolism in the stem of cut flowers, which causes abnormal flower opening, petal wilting and early senescence, resulting in a loss of quality and economic value (Liu et al. 2013). Many studies have investigated the processes associated with water deficit stress and the consequent effects on flower opening and senescence at the transcriptional and post-translational levels (Fig. 2). In rose, the NAC transcription factor gene, *RhNAC2*, is up-regulated after dehydration stress, and its silencing reduces the recovery of intact petals and petal discs during subsequent rehydration. Biochemical and functional analyses have also shown that *RhNAC2* is involved in regulating flower opening under dehydration stress by affecting cell expansion (Dai et al. 2012), and that the RhNAC2 protein can bind to the promoter of the expansin gene *RhEXPA4*. NAC3 may also participate in flower opening under water stress conditions and enhances the dehydration tolerance of petals by regulating the osmotic adjustment-associated genes, such as delta1-pyrroline-5-carboxylate synthase 1 (*RhP5CS1*) and glutathione S-transferase (*RhGST*) (Jiang et al. 2014). Water deficiency also increases abscisic acid (ABA) content and ethylene production in carnation flowers and shortens their longevity (Nukui et al. 2004), a process that was also observed in drought-induced senescence in daffodil (*N. pseudonarcissus*) (Hunter et al. 2004a) and daylily (Panavas et al. 1998). Interestingly, Fan et al. (2020) reported that a JA feedback loop mediated by an RhHB1/RhLOX4 regulatory module plays an important role in the dehydration tolerance in dehydrated rose flowers.

After a period of dehydration, rose flowers can quickly return to a normal petal expansion rate and fresh weight following the addition of water. During this rehydration phase, ethylene production increases rapidly and it is emitted as a transient burst by the gynoecium, coincident with a rapid increase in the expression of the protein kinase *RhMPK6*. The RhMPK6 protein phosphorylates and stabilizes RhACS1, a rate-limiting enzyme in ethylene biosynthesis. This burst in ethylene production is necessary to trigger the recovery response after a short period of rehydration. Thus, it has been proposed that the RhMPK6-RhACS1 module is central to sensing rehydration and transducing a signal to mediate ethylene regulated flower opening (Meng et al. 2014). Expression of the protein kinase *RhMKK9* is also specifically and rapidly induced by rehydration in the gynoecium, and has been shown to function upstream of, and to activate, the RhMPK6-RhACS1 cascade (Chen et al. 2017) (Fig. 2). Similar results were found in a study of carnation, where removal of the gynoecium repressed the production of ethylene and delayed petal senescence (Shibuya et al. 2000).

Aquaporins play important roles in the water stress response and in maintaining water homeostasis by controlling water transport across membranes. In rose, the expression of two aquaporin genes, *RhPIP1;1* and *RhPIP2;1*, partially inhibits petal cell expansion and affects water content during flower opening (Ma et al. 2008; Chen et al. 2013). A RhPIP2;1-RhPTM module has been found to regulate the trade-off between growth and stress (Zhang et al. 2019), and a recent study suggested that RhPIP2;1 functions as a dehydration sensor that is phosphorylated during water deficiency. This post-translational modification results in translocation of the RhPTM C terminus to the nucleus. Silencing of *RhPTM* greatly increases the expression of genes involved in starch and sucrose synthesis, including sucrose synthase 3 (*RhSUS3*) and ribulose bisphosphate carboxylase small-chain 1A (*RhRBCS1A*), and promotes petal expansion. A model reflecting dehydration and rehydration regulation of flower opening in rose is shown in Fig. 2.

#### Temperature

Different petal parts respond differently to temperature, resulting in the opening and closure movements of flowers. In *Crocus*, the optimum temperature required for cell expansion on the abaxial side is much lower than for the adaxial side. In a low-temperature environment, the growth rate of the abaxial side is greater than that of the adaxial side, resulting in petal closure and abnormal flower opening (Wood 1953). From an evolutionary perspective, differences in ambient temperature requirements for cell expansion on the adaxial and abaxial of petals is an adaptive mechanism that enables flowers to close petals when facing harsh conditions, such as cooling, precipitation and strong winds. Petal closure can protect the pistil and stamens to improve the rate of pollination (Abdusalam and Tan 2014; Franchi et al. 2014).

Temperature changes can cause petal movement by affecting petal cell turgor. In tulips (*T. gesneriana*), when the ambient temperature rises from 5 °C to 20 °C substantial amounts of water enter the petals and the flower rapidly opens; conversely, when ambient temperature drops returns to 5 °C, water flows out of the petals and the flowers close. The temperature-dependent opening and closure movement of tulip petals can be regulated by reversible phosphorylation of a plasma membrane aquaporin (PM-AQP) (Azad et al. 2004), which leads to an activate water channel composed of PM-AQP subunits. In contrast, in a low-temperature environment (5 °C), dephosphorylation of PM-AQP causes the inactivation of this water channel. Treatment with a calcium ion chelator and calcium ion channel blocker was reported to inhibit flower opening caused by higher temperature, indicating that calcium-dependent protein kinases (CDPKs) are involved in regulating water transport and flower opening and closure by phosphorylating PM-AQP proteins (Azad et al. 2004, 2007, 2008).

#### Dark-light cycle

Many ornamental flowering plants, such as *E. grandiflorum*, *N. tetragona*, *T. cordata*, *E. oxypetalum*, *Nymphaea* and *Hemerocallis,* show a diurnal rhythm of oscillating flower opening and closure (Bai and Saneyuki 2015; Ren et al. 2019). Time-lapse photography has been used to record flower opening in many species and it has been shown, in species such as morning glory (*I. nil*), Asiatic Lily (*Lilium hybrida*), and *E. grandiflorum*, that the time of flower opening is closely synchronized with the dark-light cycle (Bieleski et al. 2000b; Shinozaki et al. 2014; Bai and Saneyuki 2015). Other studies have indicated that flower opening is independent of light conditions, but is instead regulated by an internal circadian clock (Yon et al. 2016). In *Nicotiana attenuate*, silencing of the *Late Elongated Hypocotyl* (*LHY*) and *ZEITLUPE* (*ZTL*) genes which are known to be core clock components alters the internal rhythm, resulting in corolla opening and flower movement (Yon et al. 2016).

#### Carbohydrates and nutrients

Carbohydrate content is another environmental factor influence flower opening. In most plants, carbohydrates required to drive flower opening may derive from stored and/or imported carbohydrates (van Doorn and van Meeteren 2003). Young petals in some species, such as *Lilium* (Bieleski et al. 2000a) and *Alstroemeria* (Collier 1997), contain a large quantity of starch which is rapidly converted to glucose and fructose when flower opening. To date, most studies on the effects of carbohydrates on flower opening are at the physiological level, while the molecular mechanisms are rarely reported.

Nutrient status also affects postharvest flower opening. Among all the macroelements in nutrient solutions, calcium has the most beneficial effect on the retention of flower quality (Torre et al. 2001; Banijamali et al. 2018). In rose, CaCl_2_ treatment promoted bud opening and extended longevity (Halevy et al. 2001). Cytosolic calcium is considered to be second messenger in regulation of important cellular events, but the regulatory mechanism of calcium signaling on flower opening is still unclear. Therefore, molecular mechanism of nutrients influencing flower opening still needs further study.

## Regulatory mechanisms underlying flower senescence

### Flower senescence is an irreversible, highly coordinated process

Flower senescence generally refers to the process of programmed cell death that is associated with loss of petal function, and can be divided into two types: wilting senescence and abscission senescence. Flowers are a mixture of vegetative and reproductive organs. The main flower organs, the petals, are not directly involved in reproduction, but their function is to attract pollinators. Petals remain a ‘sink’ until fertilization and fruit set, then during senescence catabolic processes are activated and the petals convert into a ‘source’, although their nutrient recycling rate is much lower than that of leaves (Jones 2013). Petal senescence is also accompanied by orderly disintegration of internal cell structure, degradation of macromolecules and membrane systems, and recycling of various compounds. It is an irreversible process with several features that are unique to petals: 1) there are almost no chloroplasts in petal cells, while most contain chromoplasts; 2) within the petal metabolome there are low levels of few energy storage or transport compounds, such as carbohydrates, and most are secondary metabolites such as anthocyanins, carotenoids, and volatile components; 3) sugar levels in petals usually continuously decrease during senescence (van Doorn and Kamdee 2014). As is the case in fruit, petal senescence can broadly be divided into two types: ethylene sensitive and ethylene insensitive (Rogers 2013).

Senescence is the terminal stage of petal development and involves color change, loss of fragrance, wilting and shedding (Fischer 2012). Petal color in most flowering species fades during senescence, although in some the petals become blue (Schmitzer et al. 2010). In rose, flavonoid and anthocyanin levels increase in petals during senescence, and consequently the petal color develops to a deep blue shade (Lv et al. 2014). Unlike changes in morphology and color, floral fragrance in some species is not lost at the onset of senescence and volatiles emission continues for a period (Paul et al. 2019). This may be an advantage in prolonging the attraction of pollinators to other flowers of the same species.

### Phytohormones orchestrate flower senescence

#### Ethylene

**Fig. 3:**
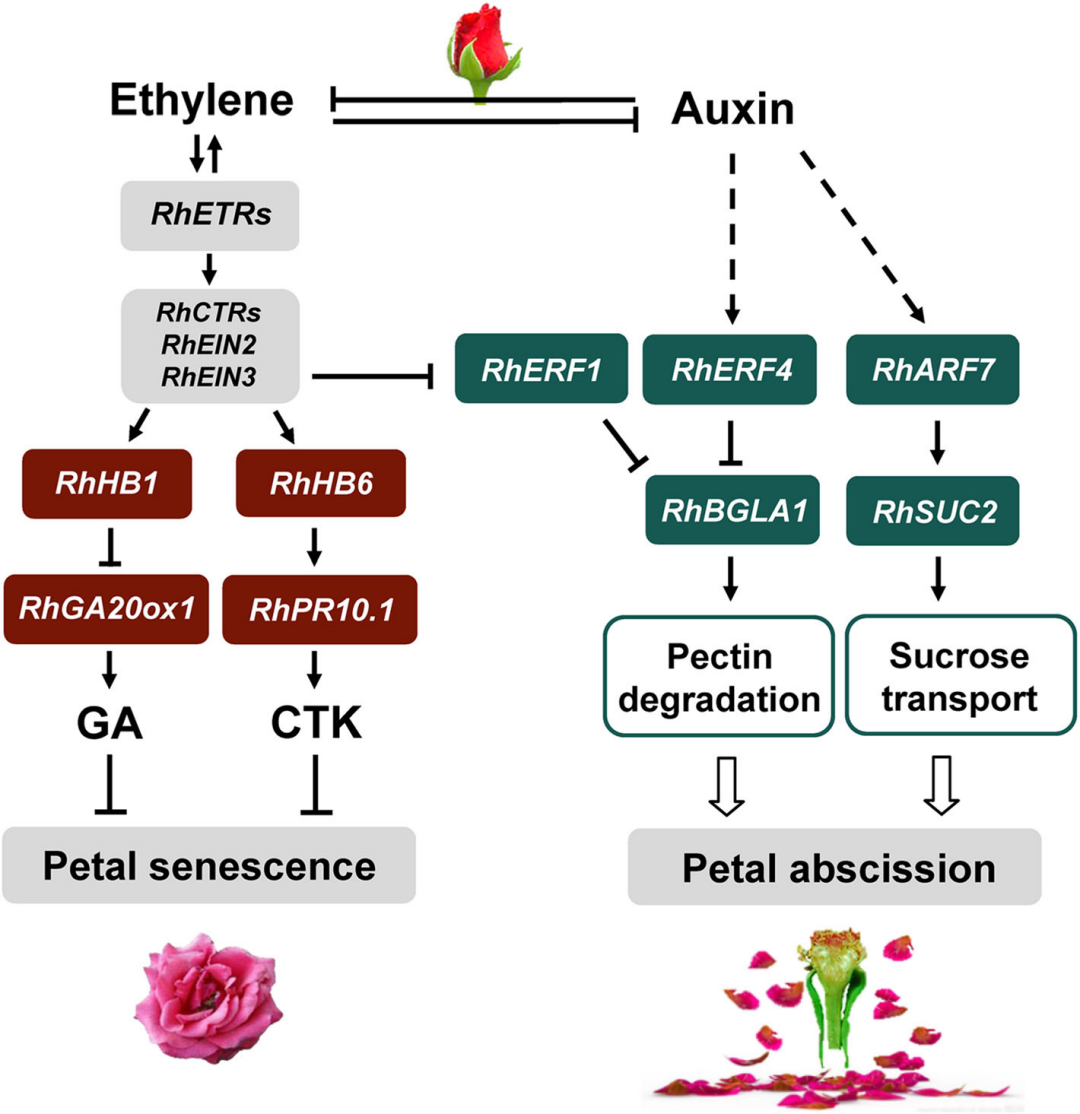
Hormonal crosstalk during rose flower senescence

Ethylene is regarded as a centrally important phytohormone in the regulation of flower senescence, and has been shown to accelerate flower senescence in many ethylene sensitive ornamental plants (Ma et al. 2005; Ichimura et al. 2009). In most ethylene sensitive flowers, such as petunia and carnation, petal senescence is also accompanied by a rapid increase in ethylene production, which is similar to the positive feedback regulation of ethylene biosynthesis in climacteric fruits (Wang et al. 2018; Shibuya et al. 2002). However, in some ethylene sensitive flowers, such as rose and peony (*P. suffruticosa*), ethylene can promote the senescence of petals, but there is no typical positive feedback regulation of ethylene biosynthesis (Ma et al. 2005). Ethylene production in the stigma is thought to trigger the expression of ethylene biosynthetic genes, 1-aminocyclopropane-1-carboxylic acid synthesis genes (ACSs) and ACC oxidase genes (ACOs), in the onset of flower senescence (Ma et al. 2018). Ethylene can promote petal senescence through the induction of RhETR3 and the signal transduction *RhCTR* genes (Ma et al. 2005, 2006; Tan et al. 2006) (Fig. 3). Thus, there may be different modes of ethylene response and regulation in ethylene sensitive flowers. Regarding ethylene insensitive flowers, little is known about the role of ethylene in them, and more studies are needed to elucidate ethylene independent pathways (Reid and Jiang 2012).

#### Auxin

Auxins have been found to delay senescence of some cut flowers, such as carnation and petunia (Halevy and Mayak 1981). Interestingly, in carnation the expression of Auxin/indole-3-acetic acid (*Aux/IAA*) transcription factor genes increases briefly in senescing petals, while the opposite is seen in *M. jalapa* (Xu et al. 2007; Price et al. 2008). Furthermore, the levels of endogenous auxins do not change during flower senescence in *L. longiflorum*, *Ranunculus*, *Ipomoea* and *Hemerocallis* flowers (Lombardi et al. 2015). Auxin is also associated with abscission-type of senescence. In rose, an *Aux/IAA* gene, *RhIAA16*, is upregulated in response to petal shedding and down-regulation of *RhIAA16* accelerates petal abscission (Gao et al. 2016). The regulation of petal abscission is complex and involves a balance between auxin and ethylene signaling pathways. Expression of *RhERF1* is inhibited by ethylene whereas *RhERF4* is induced by auxin, and both *RhERF1* and *RhERF4* directly regulate expression of *β-GALACTOSIDASE 1* (*RhBGLA1*), which encodes a pectin-metabolizing enzyme. A reduction in *RhBGLA1* expression results in less pectin degradation and delayed petal abscission (Gao et al. 2019) (Fig. 3). A recent study found that auxin regulates the gene encoding the signaling protein RhARF7, which can bind to the promoter of the *RhSUC2* sucrose transporter. Silencing of *RhSUC2* or/and *RhARF7* results in a reduction in sucrose levels in petals and promotes petal abscission. Fig. 3 shows the regulation of auxin-modulated sucrose transport during petal abscission by the RhAFR7-RhSUC2 module (Liang et al. 2020).

#### Cytokinin (CTK)

CTK is plays a critical role in delaying leaf senescence (Zwack et al. 2013), and the exogenous application of active CTKs is a widely used horticultural practice to delay plant senescence. In dahlia (*Dahlia hybrida*), treatment with the synthetic CTK 6-benzylaminopurine (BA) extends the vase life of cut flowers (Shimizu-Yumoto et al. 2020), and in *Lilium*, exogenous CTK can prolong flower life, but only if applied at the beginning of flower opening, since sensitivity to CTK gradually decreases after flower opening (Cubría-Radío et al. 2016). A CTK analogue thidiazuron dramatically extends flower longevity in ethylene insensitive *Iris* (Macnish et al. 2010). In rose, petunia and carnation, the CTK level is negatively correlated with the flower senescence process (Taverner et al. 1999; Chang et al. 2003; Wu et al. 2017). Recently, it was found that a microRNA inhibits CTK biosynthesis by downregulating expression of isopentenyl transferase (IPT) genes to promote leaf senescence (Zhang et al. 2020). Overexpression of *IPT*, driven by the *SAG12* promoter, in petunia led to increased CTK levels in petals and extended flower longevity (Chang et al. 2003). In rose, silencing of *RhPR10.1* resulted in lower CTK content, downregulation of the expression of three CTK signaling pathway genes, and accelerated petal senescence. The RhHB6-RhPR10.1 regulatory module antagonizes ethylene induced flower senescence by regulating the CTK content (Wu et al. 2017) (Fig. 3).

#### Other hormones

GA has anti-senescence effects and its exogenous application prolongs the vase life of cut flowers such as *Hemerocallis* and *Iris* (Hunter et al. 2004b; van Doorn and Woltering 2008). In addition, GA delays flower senescence in carnation, depending on the stage of flower development (Saks et al. 1992). Crosstalk between GA and ethylene can also affect flower senescence: in carnation, for example, GA can delay flower senescence through modifying the release of ethylene (Saks et al. 1992). In rose, the protein encoded by the ethylene-induced gene *RhHB1* inhibits the expression of a key GA synthesis enzyme, *RhGA20ox1*, thereby reducing GA levels and promoting petal senescence (Lv et al. 2014) (Fig. 3).

Exogenous ABA treatments have been shown to accelerate flower senescence in some plants, including *Hemerocallis* and *Gladiolus grandiflora* (Panavas et al. 1998; Kumar et al. 2013), and in ethylene-sensitive flowers, such as carnation, ABA promotes petal senescence and wilting by increasing ethylene production (Mayak and Dilley 1976). The accumulation of ABA can be inhibited by blocking ethylene signal transduction during petal senescence (Ronen and Mayak 1981) and, conversely, ethylene-induced *PhHD-ZIP* expression induces the expression of ABA biosynthetic genes and promotes petal senescence in petunia (Chang et al. 2014).

### Pollination is a key factor influencing flower senescence

Pollination results in a series of physiological and biochemical changes that often cause petal senescence. In many ethylene-sensitive flowering plants, including petunia, *Eustoma*, carnation, and orchid, pollination is accompanied by an increase in ethylene production, and there is also a burst in ethylene synthesis shortly after fertilization (Larsen et al. 1995; Halevy 1998; Xu and Hanson 2000). Pollination can obviously shorten the vase life of cut *Eustoma* (Shimizu-Yumoto and Ichimura 2006). In carnation, the ethylene precursor ACC produced from the pollinated stigma is translocated, via the style and ovary, to the petals. There, it up-regulates the expression of ethylene biosynthetic genes and induces the production of ethylene, accelerating petal senescence (ten Have and Woltering 1997). In gentian (*Gentiana scabra*), pollination significantly increases ethylene production of gynoecium, stamens and petals and promotes petal senescence (Shimizu-Yumoto and Ichimura 2012). Besides, pollination induces autophagy in petals via ethylene. In petunia, pollination induces the expression of *autophagy-related gene 8* (*ATG8*) which is regulated by ethylene (Shibuya et al. 2013). Silencing of autophagy genes, *PhATG6* and *Phosphoinositide 3-Kinase* (*PhPI3K*), accelerates petal senescence in petunia, thereby reducing flower longevity (Lin and Jones 2021).

## Perspectives

The opening and senescence of flowers have evolved in angiosperms to enhance survival and reproduction. Flower opening, as a result of coordinated movements between flower organs, exposes stamens to attract pollinators and complete pollination (van Doorn and van Meeteren 2003). Many studies have shown that pollination can activate or promote petal senescence in some species, and this process is regulated by developmental signals. Thus, flower opening and senescence are closely linked during plant development (Stead 1992; Xu and Hanson 2000).

Given the diversity of flower morphology, it is not surprising that flower opening mechanisms also vary. However, at present much remains to be learned about the specific regulatory events involved. Petal movement is mainly driven by differences in cell expansion in different parts of the petal (Liang and Mahadevan 2011). The external environment can cause a series of changes in endogenous signals within the flower, and much research has focused on the influence of environmental factors and phytohormones on the asymmetric growth driving petal movement. A promising area of future research is the nature of the interactions between environmental factors and phytohormones. For example, is there a signal cascade amplification process that stimulates hormone levels or other regulatory factors, and do the signals triggered by the external environment and hormones act synergistically?

Another research hotspot is the initiation of petal senescence. Some studies have suggested that endogenous developmental signals and the precise balance between hormone synthesis and induction during development regulate senescence initiation, which may also involve epigenetic modifications (Woo et al. 2019). To elucidate the regulatory mechanisms of petal senescence initiation, potentially important approaches include: an investigation of petal senescence mechanisms initiated by developmental signals; the characterization of the effect of changes in hormone levels on the initiation of petal senescence; and the identification of coordinators that integrate developmental and hormone signals during petal senescence. Two other important areas also should be revealed: what are molecular regulatory mechanisms underlying the differences between 1) wilting and abscission types, and 2) ethylene sensitive and insensitive types.

Gene editing technology has emerged as a rapid and effective tool for modifying plant processes and the use of CRISPR/Cas9 technology will undoubtedly become widely used in plant molecular breeding research and functional verification of genes (Schindele et al. 2020; Wei et al. 2020). The advantage of the CRISPR technology is that it can generate true single gene knockout mutants or mutants in multiple genes, both of which can help elucidate the complex regulatory networks involved in flower opening and senescence. Flower development including opening and senescence sits at the intersection of esthetic and commercial spheres of society and the physical and life science fields. Multiple disciplines and technologies, including imaging, computer simulation and fluorescence tracking will all contribute to the development of models of flower development in different species and a greater understanding of this dynamic process.
